# Cell Suspension Culture-Mediated Incorporation of the Rice *Bel* Gene into Transgenic Cotton

**DOI:** 10.1371/journal.pone.0039974

**Published:** 2012-07-02

**Authors:** Liping Ke, RuiE Liu, Bijue Chu, Xiushuang Yu, Jie Sun, Brian Jones, Gang Pan, Xiaofei Cheng, Huizhong Wang, Shuijin Zhu, Yuqiang Sun

**Affiliations:** 1 College of Life and Environmental Science, Hangzhou Normal University, Hangzhou, Zhejiang, China; 2 College of Agronomy, Shihezi University, Shihezi, Xinjiang, China; 3 Faculty of Agriculture, Food and Natural Resources, The University of Sydney, Sydney, Australia; 4 College of Agriculture and Biotechnology, Zhejiang University, Hangzhou, Zhejiang, China; Kansas State University, United States of America

## Abstract

Cotton plants engineered for resistance to the herbicides, glyphosate or glufosinate have made a considerable impact on the production of the crop worldwide. In this work, embryogenic cell cultures derived from *Gossypium hirsutum* L. cv Coker 312 hypocotyl callus were transformed via *Agrobacterium tumefaciens* with the rice cytochrome P450 gene, *CYP81A6* (*bel*). In rice, *bel* has been shown to confer resistance to both bentazon and sulfanylurea herbicides. Transformed cells were selected on a liquid medium supplemented alternately or simultaneously with kanamycin (50mg/L) and bentazon (4.2 µmol). A total of 17 transgenic cotton lines were recovered, based on the initial resistance to bentazon and on PCR detection of the *bel* transgene. *Bel* integration into the cotton genome was confirmed by Southern blot and expression of the transgene was verified by RT-PCR. In greenhouse and experimental plot trials, herbicide (bentazon) tolerance of up to 1250mg/L was demonstrated in the transgenic plants. Transgenic lines with a single copy of the *bel* gene showed normal Mendelian inheritance of the characteristic. Importantly, resistance to bentazon was shown to be stably incorporated in the T1, T2 and T3 generations of self-fertilised descendents and in plants outcrossed to another upland cotton cultivar. Engineering resistance to bentazon in cotton through the heterologous expression of *bel* opens the possibility of incorporating this trait into elite cultivars, a strategy that would give growers a more flexible alternative to weed management in cotton crops.

## Introduction

Cotton (*Gossypium hirsutum* L.) has long been the world’s most important source of natural textile fiber. Although the cotton plant is susceptible to a wide range of environmental stressors, over 180 million people worldwide depend on it for their livelihood. Many advances have been made through traditional selection and breeding programs. Biotechnologies have now been introduced into these programs. Cotton was one of the first cultivated crops to include transgene-mediated herbicide tolerance (Ht). Weed control based on the application of herbicides to eliminate susceptible weeds within a herbicide-resistant transgenic crop has been widely adopted by growers [1], [2], [3]. Herbicide-resistant transgenic crops have now been generated in a number of species for non-selective herbicides such as glyphosate, bromoxynil, triazolopyrimidine sulfonanilides, imidazolinones and 2,4-dichlorophenoxyacetic acid (2,4-D) [1], [3]. A flexible approach is vital for any weed management program and, amongst other benefits, the development of new herbicide-tolerant crops would minimize the potential for the evolution of tolerance to the herbicides currently used with Ht transgenic lines. In this paper, we describe the novel integration into cotton of a rice gene, *CYP81A6* (*bel*), which has been shown to confer resistance to the herbicides, bentazon and sulfonylurea.

Bentazon, known commercially as Basagran, is a benzothiadiazinone contact herbicide that controls broadleaf weeds and sedges by disrupting photosystem II electron transfer [4], [5], [6], [7]. Resistance to bentazon in non-susceptible species is primarily based on metabolic detoxification, mediated by oxidative reactions that are catalyzed by cytochrome P450 mono-oxygenases [8], [9], [10]. Sulfonylurea herbicides act by inhibiting the essential enzyme, acetolactate synthase. The herbicide resistant, *CYP81A6*, *bel* gene was originally cloned from two rice bentazon lethal mutants, *bel* ^a^ and *bel* ^b^, that were more susceptible to the herbicide than wild-type plants. The bentazon- and sulfonylurea-lethal phenotypes of the mutants were the result of defects in the ability of the plants to metabolize these xenobiotics [11].

Cytochrome P450 monooxygenases are NADPH-dependent heme proteins that code for a large and diverse group of isozymes that mediate a wide range of oxidative reactions in plants, animals, and microorganisms [12], [13], [14], [15]. In plants, cytochrome P450s are known, for example, to mediate the biosynthesis of lignins, terpenoids, alkaloids, sterols, fatty acids, and many plant defense-related secondary compounds [14], [15], [16]. Several plant P450s have been shown to be capable of metabolizing herbicides to harmless metabolites. These include the *bel* gene, the *CYP71A11* and *CYP81B2* genes from tobacco [17], *CYP71B1* from *Thlaspi arvensae* [18], *CYP71A10* from soybean [19], and *CYP73A1* [20], *CYP76B1* [21], and *CYP81B1* [22] from *Helianthus tuberosus*. All, apart from *bel*, are known to detoxify chlortoluron [17], [18], [19], [23].

**Figure 1:**
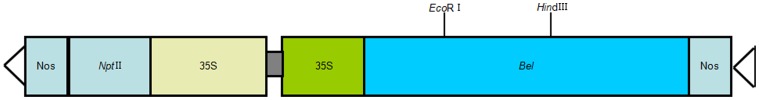
Schematic diagram of T-DNA incorporating the *Bel* expression cassette and the *npt* II selective marker gene.

Traditional breeding programs for commercial cotton fiber production are based on the upland cotton species, *Gossypium hirsutum* L. These breeding programs have led to a steady improvement in agronomic traits. However, a paucity of useful economic characteristics in the breeding stock and the availability of potentially useful genes in other organisms have led to the incorporation of genetic transformation technologies into cotton breeding programs [24], [25], [26]. Although several genetically modified cotton lines have been widely adopted, difficulties with the transformation and regeneration of cotton remains an impediment to its widespread adoption. A number of improvements to the transformation procedure have been published that increase transformation efficiency and reduce somaclonal variation. A reliable and effective step-by-step protocol that incorporates numerous improvements and modifications was published by Wilkins et al. (2004) [25]. We have refined the protocol for cotton transformation further to improve its efficiency and reducing the time required to develop new transgenic lines. Through the use of our protocol, we have developed transgenic cotton lines heterologously expressing the rice *bel* gene, that confers resistance to the herbicide, bentazon. This provides the potential for further flexibility for weed management in cotton crops.

**Figure 2:**
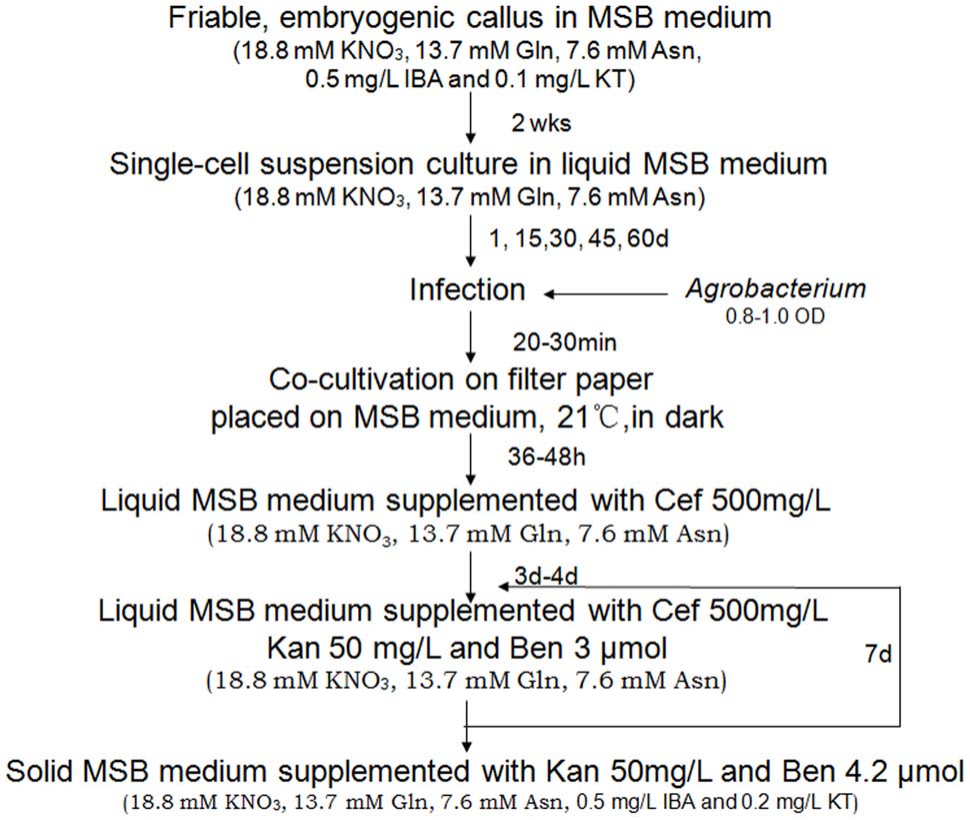
Scheme for *Agrobacterium*-mediated transformation of suspension cultures and plant regeneration in upland cotton.

## Materials and Methods

### Plant Materials

*G. hirsutum* L. Coker 312 seeds were de-coated and sterilized by dipping in 70% (v/v) ethanol prior to a 10 min exposure to 0.1% (w/v) HgCl_2_. They were subsequently rinsed in sterile distilled water and germinated on 1/2 MS (Murashige & Skoog medium) [27] medium with 10% (w/v) glucose and 0.25% (w/v) Phytagel (Sigma, USA) at 28°C in the dark for 3 d and transferred to the culture room (28°C, 14-h photoperiod, irradiance of 135 µmol m^–2^s^–1^ provided by cool-white fluorescent lamps) for 4 days.

Hypocotyls were excised from aseptic seedlings and cut into 5–7 mm segments. Callus induction was carried out on MSB medium [MS inorganic salts and B_5_ vitamins [28]] supplemented with 3% (w/v) glucose, 0.25% (w/v) Phytagel, 0.1 mg/L 2,4-dichlorophenoxyacetic acid (2,4-D)(Sigma, USA), and 0.2 mg/L kinetin (Sigma, USA) for 4–6 weeks. Embryogenic callus was induced on MSB medium supplemented with 18.8 mM KNO_3_, 13.7 mM glutamine, 7.6 mM asparagine, 3% (w/v) glucose, 0.25% (w/v) Phytagel, 0.5 mg/L Indole-3-butyric acid (IBA) (Sigma, USA), and 0.1 mg/L kinetin. After subculturing for 12 to 15 days, embryogenic calluses were collected and inoculated into 30 ml of liquid MSB medium in 100-ml Erlenmeyer flasks containing 18.8 mM KNO_3_, 13.7 mM glutamine, 7.6 mM asparagine, and 3% (w/v) glucose, in order to establish suspension cultures for transformation (15 to 60 days). The liquid medium was removed every 7 days and replaced with 30 ml of fresh liquid medium.

### Bacterial Strains and Plasmids

The *Agrobacterium tumefaciens*-disarmed helper strain LBA4404 [29], harbouring the plasmid pC450-2 that carries p35SCYP81A6 and the *npt*II gene as the selectable marker and was used for transformation and regeneration experiments. CYP81A6 was cloned from rice by Pan et al (2006) [11] (Figure 1) and had been shown to confer resistance to the bentazon and sulfonylurea herbicides. The binary vector was transferred to *Agrobacterium* by the heat-shock method [30]. The transformed *Agrobacterium* was grown on LB medium plates containing rifampicin (25 mg/l) and kanamycin (50 mg/l) (Sigma, USA) (LB medium [31]: Bacto Tryptone 10 g/L, Bacto Yeast Extract 5 g/L and NaCl 10 g/L). Five single colonies were inoculated individually in 20 ml LB medium containing rifampicin (25 mg/l) and kanamycin (50 mg/l) in flasks and these were grown for 36 h at 28°C with 200 rpm shaking. Another 10 ml of MGL medium (Tryptone 5 g/L, NaCl 5 g/L, MgSO_4_.7H_2_O 0.1 g/L, KH_2_PO_4_0.25 g/L, Gly 1.0 g/L, pH 5.8) was added to the *Agrobacterium* suspension for *Agrobacterium* activation on a shaker at 200 rpm to A6_00_ 1.0–1.5 for 2–3 h at 28°C in a 100 ml flask. Just prior to co-cultivation, the *Agrobacterium* were diluted by fresh MGL medium to OD_600_ to 0.8–1.0. Additional acetosyringone (AS) (pH 5.8) was added to the culture at a final concentration of 100 µM.

### Co-cultivation

One, 15, 30, 45 and 60 day-old suspension cultures were prepared for infection by *Agrobacterium*. Friable embryogenic calluses was cultured in liquid MS medium for one day was also prepared for infection and used as a control. 3-4 day old post-subculture embryogenic cell suspension cultures growing on liquid MSB medium with IBA (0.5 mg/l) were used for transformations. The process of *Agrobacterium*-mediated transformation of suspension cultures is shown in Figure 2. Somatic cell suspension cultures were collected by sterile pipette and sieved through a 100 µm filter to remove large cell clumps and then desiccated for 15–20 min. The suspension cultures were incubated with *Agrobacterium* culture LBA4404 (OD_600_ 0.8–1.0) for 30 min. The suspension cultures were then transferred onto sterile filter paper and placed on the solid co-culture medium (CM medium, MSB medium, 0.25% (w/v) phytagel, 3% (w/v) Glucose) for 36 to 48 hours in the dark at 21°C. 1 ml of 50 mg/L As was added to the sterile filter paper. Three flasks of suspension cultures with different culture times (1, 15, 30, 45 and 60 day, respectively) were co-cultivated with *Agrobacterium* with OD_600_ value of 0.8–1.0. The co-cultivation with cultures of different ages was repeated three times and the number of bentazon-resistant colonies per flask was determined.

After co-cultivation, the cells along with the filters were transferred to sterile water supplemented with cefotaxime (200 mg/l) (Sigma, USA) and washed 2-3 times. The cells were then cultured in liquid MSB medium supplemented with cefotaxime (500 mg/l) for 3-4 days. The liquid medium was then removed and the same liquid medium supplemented with kanamycin (50 mg/l) and bentazon (3 µM) (Sigma, USA) was applied. The cultures were then incubated at 28°C at 110 rpm on a rotary shaker. The liquid medium was changed every 7 days for 4 times to eliminate the false positive cells and proliferate the resistant cells, when no more *Agrobacterium* contamination appeared, the cefotaxime was eliminated from the medium. The suspension cells were then transferred to solid MSB medium supplemented with kanamycin (50 mg/l) and bentazon (4.2 µM) for proliferation.

### Inducing Kanamycin and Bentazon Resistant Callus

After the first selection in liquid suspension culture, cell suspension cultures were again transferred to a solid selection medium (MSB, 3% (w/v) Glucose, 400 mg/L Ticarcillin, 50 mg/L Kan or/and Ben 4.2 µmol, 0.5 mg/L IBA and 0.2 mg/L KT, 0.3 (w/v) % Phytagel) for 3-4 weeks. Proliferation of Kan and Ben-resistant calluses was subcultured on the same solid media.

**Figure 3:**
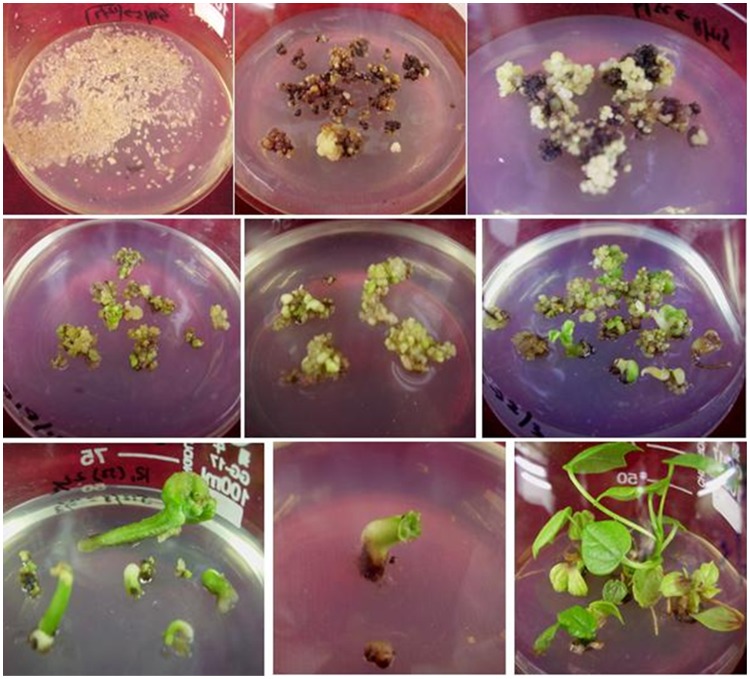
Formation of transformed callus resistant to Bentazon and Kanamycin and transgenic plant regeneration via somatic embryogenesis. A. Infected suspension cultures on solid medium, B. Cell masses browning and new callus formation, C. New somatic embryogenic callus formation, D–E. Callus proliferation and somatic embryogenesis from different colonies, F–H. Somatic embryo formation, maturity, I. Cotton plant regeneration on medium supplemented with Kanamycin and Bentazon.

**Table 1:** The percentage of transformed colonies resistant to Bentazon and Kanamycin according to duration of suspension culture.

time	1 day			15 days			30 days			45 days			60 days		
R-colonies1	4	2	3	13	12	8	11	9	8	15	22	19	14	16	21
R-colonies2	5	2	–	12	9	10	13	10	8	17	20	19	23	18	20
R-colonies3	7	–	3	7	11	–	–	14	12	18	16	21	19	17	15
Average	3.7			10.3			10.6			18.6			18.1		

R-colonies represent the number of colonies resistant to Bentazon and Kanamycin from the infected suspension cultures in one bottle, the transformation was repeated 3 times with different suspension cultures (1, 15, 30, 45 and 60 day-old); – represents callus colonies polluted by *Agrobacterium*; data were counted from each bottle.

**Figure 4:**
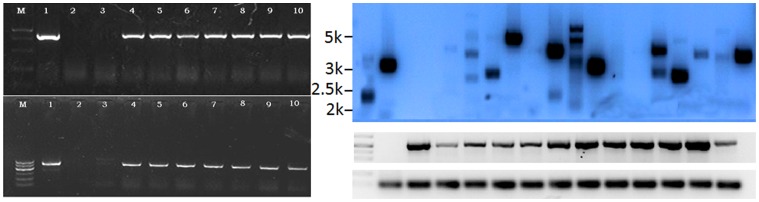
PCR and Southern blot analysis of transgenic cotton. A. PCR-based verification of stable insertion of the *npt II* gene into the genomic DNA of cotton using an *npt II -*specific probe; and B, of the *bel* gene with a *bel-*specific probe, C. Southern blot of T1 plants, Lane 1 shows positive control plasmid DNA, D. *Bel* expression analysis of RT-PCR of six plants of individual T2 and T3 plants, using *actin* as internal control. (Lane 1 showing Coker 312 as a negative control, Lane 2–7 showing T2 plants, Lane 8–13 showing T3 plants generated from T2 plants from plants in Lanes 2–7; *Actin* gene in cotton as reference gene).

**Figure 5:**
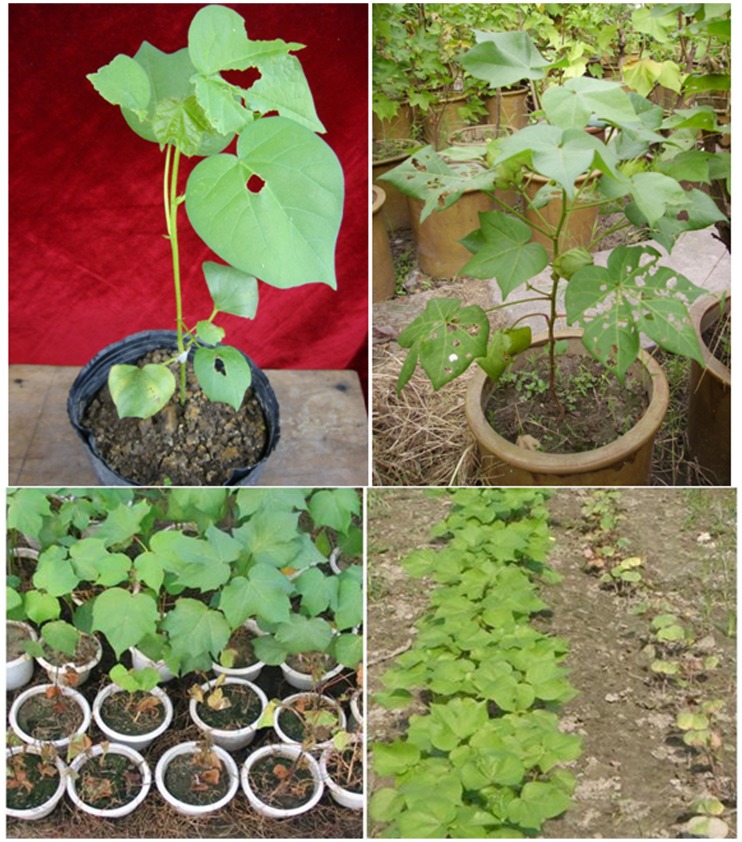
Transgenic cotton plants in pots, greenhouse and experimental plot. A. Transgenic plants grafted onto rootstock of Coker312 showing resistance to bentazon and rootstock showing sensitivity to bentazon (Arrow shows wilting 2 days after spraying with Bentazon), B. T0 transgenic cotton plant in greenhouse, C. T1 generation plants showing resistance to Bentazon, and some that are sensitive to Bentazon. D. T2 plants resistant to Bentazon and non-transgenic Coker 312 plants sensitive to Bentazon in the experimental plot.

### Differentiation of Somatic Embryos and Plant Regeneration

Calluses resistant to Kanamycin and Bentazon were transferred onto solid medium (MSB, 3% (w/v) Glucose, 18.8 mM KNO_3_, 0.25% (w/v) Phytagel, Gln 1.0 g/L, Asn 0.5 g/L, IBA 0.5 mg/L, KT 0.15 mg/L) to induce the embryogenic calluses, then embryogenic calluses were transferred onto embryo differentiation medium (MSB, 3% (w/v) Glucose, 0.25% (w/v) Phytagel, Gln 2.0 g/L, Asn 1.0 g/L, IBA 0.5 mg/L, KT 0.15 mg/L) for induction and development of somatic embryos every 4 weeks. Mature embryos (cotyledon embryo and torpedo embryo) were transferred onto the filter paper on the DM medium for germination for 30 days. After root and shoot elongation, the germinated embryos were transferred to solid medium (MSB, 3% (w/v) Glucose, 0.25% (w/v) Phytagel, IBA 0.5 mg/L, KT 0.1 mg/L) to conversion into plantlets with normal roots. Then the plantlets were planted into pots containing a 1∶1:1 mixture of vermiculite, top soil, and peat, and covered to increase humidity. Plantlets were gradually exposed to ambient humidity over a two week period and then placed in the greenhouse. Regenerated plants with minimal root systems were grafted onto wild-type Coker 312 rootstock material.

### Screening and Analysis of Transgenic Plants

#### PCR and southern blot analysis

Cotton genomic DNA was extracted from young leaves using methods described by Paterson et al. (1993) [32]. Polymerase chain reaction (PCR) and Southern blotting were used to confirm the presence of the *bel* gene and the copy number in transgenic plants. PCR analysis for detection of the *npt*II gene was performed using the primers 5′- TCG GCT ATG ACT GGG CAC AAC AGA-3′ (forward) and 5′-AAG AAG GCG ATA GAA GGC GAT GCG-3′ (reverse) that amplify a section of the *npt*II gene coding region. PCR analysis for detection of the *bel* gene was performed with the primers 5′-GAA GTT CAT GCC GGA GAG-3′ (forward) and 5′-ATT GCG GGA CTC TAA TCA TA-3′ (reverse). PCR was performed in 25 µl reaction mixtures consisting of 10 × reaction buffers, 50 ng DNA templates, 15 mM MgCl_2_, 10 mM dNTPs, 50 ng of each primer and 1 unit Taq DNA polymerase in an ABI thermal cycler (Veriti 9902, USA) using the following conditions: initial denaturation at 94°C for 2 min followed by 35 cycles of denaturing at 94°C for 1 min, annealing at 55°C for *npt*II and annealing at 55°C for *bel* gene for 1 min, extension at 72°C for 3 min and final extension at 72°C for 5 min. PCR products were analyzed by gel electrophoresis on 1% (w/v) agarose gels.

Southern blot analysis was carried out according to Sambrook et al. (1989) [33] using the DIG DNA Labeling and Detection Kit (Roche, Switzerland) according to the manufacturer’s recommendations. Plasmid DNA was used as a positive control and non-transgenic Coker 312 was used as a negative control. Approximately 10 µg of total genomic DNA was digested with *EcoR* I at 37°C overnight in a total volume of 100 µl and separated by electrophoresis on 0.8% (w/v) agarose gel at 30 V for 8 h. DNAs were blotted onto charged nylon membranes (Hybond N^+^, Bedford, MA01730) and hybridized following manufacturer’s instructions of Roche kit. PCR-amplified *bel* fragments were used as a probe with boitin-labelling with the DIG Detection Kit (DIG DNA Labeling and Detection Kit, Roche, Switzerland).

In 2007, T1 transgenic cotton plant families derived from self-fertilized T0 transgenic plants were grown in a greenhouse at the Huajiachi Campus of Zhejiang University in Hangzhou, China. Bentazon (1250 mg/L) was sprayed during the seedling stage, to test for herbicide resistance of the transgenic plants. Strongly herbicide resistant lines with normal gross morphological phenotypes were self-fertilised. The copy number of *bel* transgenes inserted was determined in the T1, herbicide tolerant lines using Southern blot analysis. Mature seeds were harvested from individual high-Bentazon-resistant plants with a single-copy insertion. Homozygous transgenic cotton lines were selected by molecular and genetic analysis of T2 lines in 2008 to get T3 seeds. T2 homozygous transgenic cotton plants were crossed with an elite cultivar for hybrid selection and testing with Bentazon sprays in the experimental plot.

#### RT-PCR analysis

In order to test for the expression level of the *bel* transgene, total RNA was isolated from young leaves of untransformed control and transformed single copy T2 and T3 plants from using the RNeasy Plant Mini Kit (Qiagen, Germany) following manufacturer’s instructions. cDNA obtained from this RNA (Eppendorf, Germany) was then used as template for PCR amplification of *bel* transcripts using the primer pair: 5′ AGAAGAAGAGCATGATCGCC 3′ (forward), 5′ TGGTTCAGCAGTAGCGACAT 3′ (reverse).

PCR conditions were: 94°C for 5 min for initial melting followed by 35 cycles of amplification with each cycle consisting of the following steps: 94°C for 1 min, 56°C for 1 min and 72°C for 1 min 15 s with a final extension at 72°C for 10 min. Primers specific to the actin gene were used as internal control: 5′ CCGATGCCTTGATGAAGATT 3′ (forward), 5′ GCAGTCTCCAGTTCCTGCTC 3′ (reverse). PCR products were analyzed by gel electrophoresis on 1% (w/v) agarose gels.

## Results and Discussion

### Generation of Herbicide Resistant Transgenic Cotton Plants

Although glyphosate, glufonidase and a number of other herbicides have previously been used to effectively control dicot and monocot weeds in cotton crops, it is desirable to develop additional transgenic cotton varieties resistant to other herbicides, as no one herbicide is sufficient for an effective weed control program. In addition, the availability of transgenic lines with tolerance or resistance genes to different herbicides can reduce the danger of developing acquired herbicide resistance in weed species. We have used an improved transformation system to develop transgenic cotton lines resistant to the herbicide bentazon through the incorporation of the rice *Bel* gene into the cotton genome. *Bel* is a cytochrome P450 gene, originally identified from work conducted on bentazon and sulfonylurea-lethal rice mutants [11].

Using our protocol, embryogenic callus and embryos were formed from the cell suspension cultures after 4 weeks. After co-cultivation with *Agrobacterium*, transformed cells were selected and putative positive colonies were transferred onto solid medium (Figure 3 A) containing 50 mg/L kanamycin and 4.2 µM bentazon. Many of the cell masses proliferating on the kanamycin and bentazon selection media browned and died. However, in many instances, yellow living calluses formed from the browning cell mass. These were subcultured onto fresh selection media (Figure 3 B, C) and 5 to 6 weeks after the co-cultivation with *Agrobacterium*, multiple distinct living colonies were obtained. A total of 504 transformed colonies were obtained from four of the suspension culture treatments, with frequencies of 3.7% in the 1-day suspension cultures, 10.3% in the 15-day cultures, 10.6% in the 30-day cultures, 18.6% in the 45-day n cultures, and 18.1% in the 60-day cultures, indicating a significant improvement in transformation efficiency between 30 and 45 day cultures (Table 1). After 4 weeks of cell proliferation and differentiation on the selective media, many somatic embryos appeared on the surface of the resistant calluses (Figure 3 C, D, E). Mature embryos were transferred to fresh solid media for conversion into plantlets via somatic embryogenesis (Figure 3 F–I). In order to verify that the colonies were comprised of transgenic cells, PCR testing was undertaken for the presence of the the *npt*II and *bel* gene. Material from 17 of 29 lines tested was confirmed as containing the *npt* II and *bel* genes incorporated in the cotton genome (Figure 4 A, B). The 17 positive lines were subsequently carried forward through the cell proliferation, somatic embryogenesis and plant regeneration processes. Twenty-six primary (T0) transgenic cotton plantlets were obtained from the 17 lines and re-confirmed by PCR analysis as containing the *bel* and *npt*II transgenes. The regenerated, transgenic cotton plants were grafted as scions onto Coker 312 rootstocks. After the graft union had formed, the grafted plants were sprayed with the herbicide (Bentazon 500 mg/L) on the leaves of the scion and the cotyledons of the rootstock. The leaves of transgenic scion remained green, whereas the rootstock cotyledons wilted, indicating that the rootstocks retained sensitivity to the herbicide, while the transgenic scion maintained its acquired resistance (Figure 5 A). Grafted transgenic plants (T0) were transferred to pots in the greenhouse for further growth and self-fertilization (Figure 5 B).

The grafted T0 transgenic lines were allowed to self-fertilize and the resulting T1 plants were tested by spraying with bentazon (1250 mg/L). Surviving plants were classed as putatively bentazon-resistant. These were screened by PCR for the presence of the *npt*II and *bel* genes. The PCR screen confirmed that all of the tested plants were positive for the transgenes. Finally, the T1 plants were examined by Southern blot for determination of transgene copy number. The Southern blots indicated that 7 lines out of the 17 tested had one copy of the T-DNA inserted into the cotton genome. Other lines were shown to have two or three copies, and four of the lines showed no signals on the Southern blot (Figure 4 C). The 7 lines with a single copy of the *bel* gene were further self-fertilized to obtain T2 seeds. Resistant seedlings with one copy of the *bel* gene appeared at a ratio of approximately 3∶1 (Figure 5 C). Similarly, the T2 plants were self-fertilized to obtain T3 seeds. Testing of T2 and T3 plants showed that the bentazon resistance was carried through to these subsequent generations. RT-PCR analysis was also conducted on T2 and T3 plants from the same lines. RT-PCR results showed that all lines had high levels of expression of the *bel* gene (Figure 4 D). Importantly, hybrids between the T2 transgenic cotton lines and an elite varieties retained resistance to bentazon. Seedlings of the T2 and T3 generations of transgenics and wild-type Coker 312 were sprayed with Bentazon (1250 mg/L) for two times 3 days later at the two true leaf stage of development in the field. All of the Coker 312 plants subsequently wilted and died. The T2 and T3 seedlings, however, continued to grow normally (Figure 5 D). Similarly, the F1 hybrid seedlings between the transgenic and the elite lines retained their resistance in the face of the same herbicide treatment used for the T2 and T3 transgenic cotton lines.

In this study, we further refined the procedure for *Agrobacterium*-mediated cotton transformation, using suspension cell cultures as explants to produce transgenic cotton lines with resistance to the herbicide, bentazon. Overall, the process of transformation, from suspension cultures to transgenic cotton plants took approximately 3-5 months. Previously published cotton transformation protocols take 6-10 months for cotton in order to obtain transgenic plants [25], [34]. Wilkins et al. [25] published a detailed protocol using hypocotyls as explant material for co-cultivation with *Agrobacterium*, the protocol requires approximately 8-10 months for the production of transgenic plantlets [25]. Our improvements to the published protocols therefore make the process less time consuming. One of the difficulties has been that many of the previously published protocols have proven difficult to replicate in other laboratories. In our protocol, cell suspension cultures are used as explants material for transformation and for the selection of transgenic clones because it has the advantages of: improving the survival of transgenic colonies; decreasing the percentage of false positive resistant calluses; and reducing the occurrence of deleterious somaclonal variations that are common in material derived from solid selection media. Certainly, our method is efficient in terms of the number of transgenic lines produced. It is known that in cell suspension cultures, many of the single cells and small cell masses become growth stage synchronous. It is possible that the success of our method is at least partially due to a uniformity of cell physiology because of this synchrony [35], [36], [37]. We believe that widespread adoption of our cotton transformation protocol has the potential to significantly accelerate the generation of transgenic lines with other agronomical useful traits.

We also expect that the bentazon resistance incorporated in the lines developed in this study will be incorporated into elite line breeding programs and will eventually give growers a useful additional tool in their arsenal.
